# Beyond the heart: prevalence and implications of extra-coronary findings in coronary CT angiography: a retrospective study

**DOI:** 10.1007/s00330-025-11886-6

**Published:** 2025-08-05

**Authors:** David A. Gloor, Inga Todorski, Alan Peters, Benedikt Wagner, Tobias Gassenmaier, Justus Roos, Andreas Christe, Lukas Ebner, Adrian T. Huber

**Affiliations:** 1 Department of Diagnostic, Interventional and Pediatric Radiology, Inselspital, Bern University Hospital, University of Bern, Bern, Switzerland; 2 Department of Radiology, Octorad AG, Hirslanden, Zürich, Switzerland; 3 Department of Radiology, Linde Hospital, Hirslanden Bern, Bern, Switzerland; 4 Department of Radiology and Nuclear Medicine, Luzerner Kantonsspital, University Teaching and Research Hospital, University of Lucerne, Lucerne, Switzerland; 5 Department of Radiology, Beau-Site Hospital, Hirslanden, Bern, Switzerland

**Keywords:** Tomography (X-ray computed), Coronary vessels, Incidental findings, Pulmonary disease, Neoplasms

## Abstract

**Objectives:**

To evaluate the prevalence and clinical implications of extra-coronary findings in a large cohort of patients undergoing coronary computed tomography angiography (CCTA).

**Materials and methods:**

This retrospective study analyzed data from 3295 consecutive CCTA examinations at a single tertiary center. Radiology reports were reviewed for potentially significant extra-coronary findings. Three-year follow-up evaluation was performed via hospital records. Prevalences of confirmed significant findings were determined and extrapolated to patients lost to follow-up.

**Results:**

Extra-coronary findings were reported in 92.7% (3053/3295) of patients. Potentially significant non-cardiovascular findings were found in 25.3% (833/3295), including potentially malignant findings in 8.5% (281/3295) and significant non-malignant findings in 19.3% (637/3295). Among patients with potentially malignant findings, 40.2% (113/281) underwent follow-up, with confirmed malignancy in 28.3% (32/113). Extrapolation suggests a malignancy prevalence of up to 2.4% (95% CI: 1.8–3.2%) within the CCTA field of view, with a minimum of 1.0% if all patients lost to follow-up were assumed to have no malignancy. The most frequent significant non-malignant finding was pulmonary emphysema (10.7%; 352/3295), with extrapolated prevalence rates of 4.2% (95% CI: 2.6–5.9% for COPD and 1.8% (95% CI: 0.8–3.4%) for asthma or obstructive sleep apnea. Liver steatosis was present in 4.5% (147/3295), with an estimated prevalence of 3.1% (95% CI: 1.6–4.2%) for metabolic dysfunction-associated steatotic liver disease and 1.3% (95% CI: 0.3–2.9%) for alcohol-related liver disease.

**Conclusion:**

Extra-coronary findings are common in CCTA and carry important clinical implications. Careful management is necessary, and additional screening protocols may benefit high-risk patients.

**Key Points:**

***Question*** *How frequently do extra-coronary findings occur in coronary CT angiography, and what is their clinical impact in patients undergoing cardiac evaluation?*

***Findings*** *Clinically significant extra-coronary non-cardiovascular findings were identified in 25.3% of patients, including unsuspected malignancies and relevant pulmonary and hepatic comorbidities.*

***Clinical relevance*** *Extra-coronary findings on coronary CT angiography are common and often clinically relevant, warranting structured assessment to support timely diagnosis and multidisciplinary patient care.*

**Graphical Abstract:**

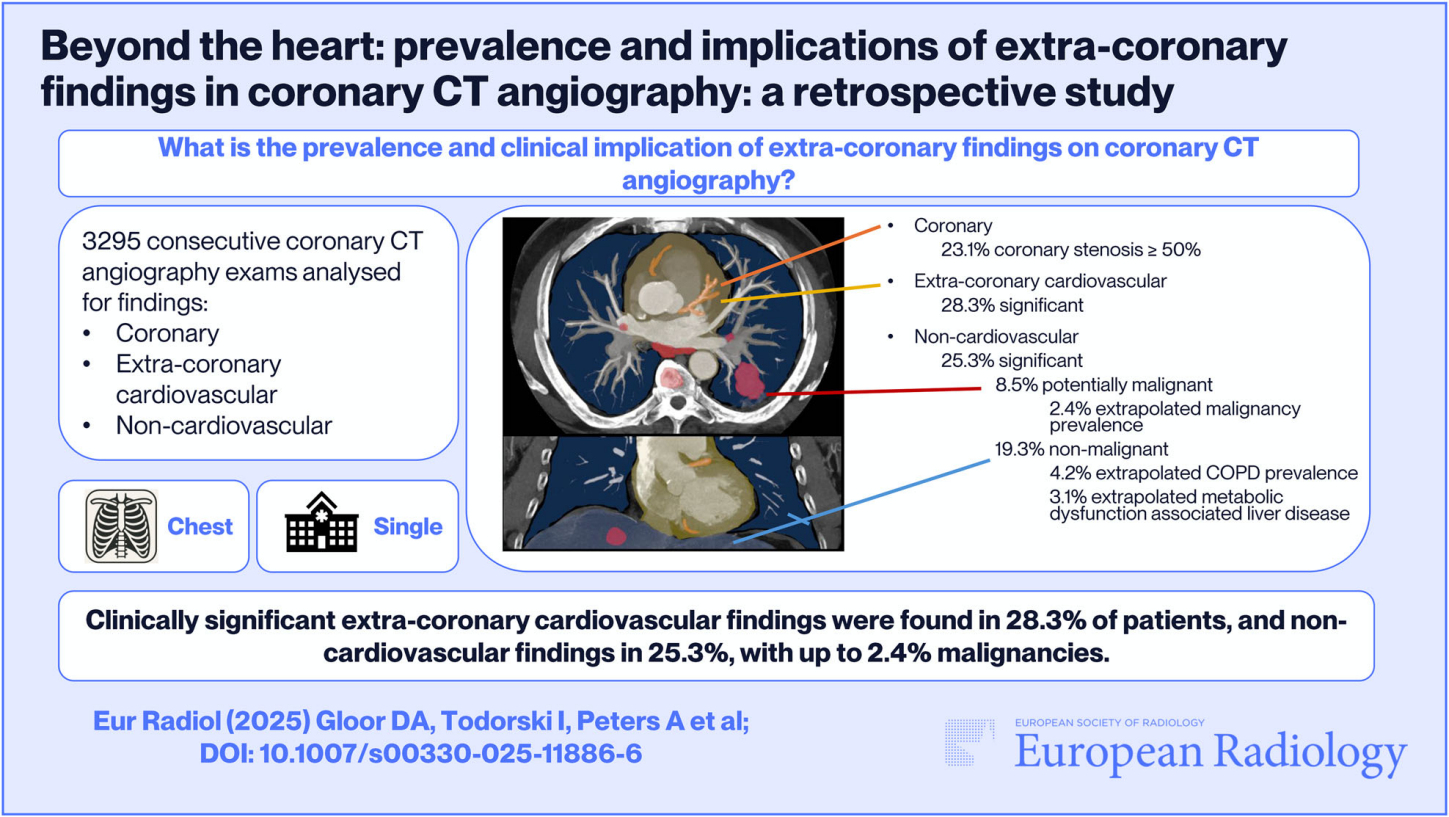

## Introduction

Over the past twenty years, cardiac computed tomography angiography (CCTA) has become the first-line non-invasive test for ruling out coronary artery stenosis in patients with low to intermediate pretest probability of coronary artery disease (CAD) [1–4]. Besides its high negative predictive value of over 90% for significant coronary artery stenosis [5], CCTA offers valuable prognostic information for non-obstructive CAD [6, 7].

Patients referred for CCTA often present with cardiovascular risk factors, such as obesity, hypertension, smoking, diabetes, and dyslipidemia [8]. These risk factors are often linked to other significant health conditions beyond the heart, such as chronic obstructive pulmonary disease (COPD) [9, 10], metabolic dysfunction-associated steatotic liver disease (MASLD) [11–13], and gastroesophageal reflux disease (GERD) [14]. Additionally, patients with cardiovascular risk factors face a higher risk for malignancies, such as lung cancer [15], esophageal cancer [16], and malignant liver lesions [17, 18]. Given the typical age range of 40–70 years [19], these comorbidities may substantially impact quality-adjusted life years (QALYs) [20]. Consequently, the detection of both coronary and extra-coronary findings during CCTA is essential for comprehensive patient care.

Previous studies have reported high rates of extra-cardiovascular findings on CCTA, with a median prevalence of 45% (range 7–100%) and potentially significant findings in 17% (range 1–67%) [21–25]. However, many of these studies lacked systematic follow-up. One earlier study found significant findings in 43% of cases, but only 6 patients had follow-up data [26]. To date and to our knowledge, there is no comprehensive data on the prevalence and clinical impact of coronary and extra-coronary findings in a large CCTA cohort. This gap is noteworthy, as CCTA populations—such as those in the SCOT-HEART trial (mean age 57, 53% smokers) [27]—closely resemble cohorts in lung cancer screening trials like NELSON (mean age 58) [28]. Notably, the NELSON trial reported similar 10-year mortality from cardiovascular disease and lung cancer. With a lung cancer detection rate of 0.9%, and a shared risk profile, systematic assessment of lung nodules and other extra-coronary findings in CCTA could enable opportunistic screening, especially among smokers.

We hypothesized that clinically significant extra-coronary findings are frequent within the CCTA scan range and that the prevalence of lung cancer may be comparable to large screening cohorts. This study aimed to evaluate their prevalence and clinical implications in a large CCTA cohort.

## Methods

### Baseline population

**Fig. 1:**
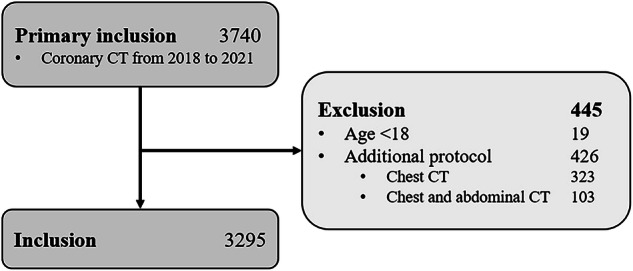
Flowchart of patient inclusion and exclusion. A total of 3740 patients underwent coronary computed tomography angiography (CCTA) between January 2018 and December 2021. After excluding 445 patients due to additional imaging or age under 18 years, 3295 patients were included in the final analysis

This retrospective study included 3740 consecutive patients undergoing CCTA at a single tertiary center (University Hospital Bern, Switzerland) between January 1, 2018, and December 31, 2021. Written informed consent was obtained; the study was approved by the Bern cantonal ethics committee (BASEC-ID 2022-00724) and conducted in accordance with the Declaration of Helsinki. The authors had full access to and took full responsibility for the integrity of the data. After excluding patients with additional imaging beyond the standard scan range (e.g., whole chest CT, abdominal CT), patients < 18 years, and those with multiphase cardiac acquisitions, the final cohort comprised 3295 patients (Fig. 1).

### CT acquisition protocol

All CCTAs were acquired on a dual-source CT scanner (Siemens Somatom Definition Flash CT, Siemens Healthineers), enabling the use of high-pitch “Flash” acquisition mode. In all patients, a non-contrast-enhanced scan of the heart for calcium scoring was followed by a contrast-enhanced scan in inspiratory breath hold. Heart rate was optimized with intravenous beta blockers (metoprolol), and coronary arteries were dilated by sublingual administration of nitroglycerin spray. All acquisitions were electrocardiogram-triggered, if feasible by using a prospectively triggered acquisition with a high pitch of 3.2. In patients with arrhythmic or high heart rates despite premedication, or insufficient prospectively triggered image quality, a retrospectively triggered acquisition was performed with a trigger range from 30% to 80% in the RR-interval. Both prospective and retrospective acquisitions were analyzed at 80% of the RR-interval.

All scans were acquired with a collimation of 128 × 0.6 mm and a gantry rotation time of 0.28 s. Non-contrast scans were performed for calcium scoring with 120 kVp and 80 mAs, whereas contrast-enhanced scans were acquired with 120 reference kV and 250 reference mAs by using the care dose mode (Siemens Healthineers). Intravenous contrast medium was injected with a CT contrast media delivery system (Exprès; Bracco Diagnostics). Contrast-enhanced scans were acquired after weight-adapted injection of 60 to 100 mL of contrast agent (Ultravist 370; Bayer Healthcare) into the left brachial vein with a flow rate of 4.5 mL/s, followed by a 20-mL saline chaser with a flow rate of 4.5 mL/s. For the contrast-enhanced scans, bolus-tracking was used, triggered on the ascending aorta starting at 70 Hounsfield units.

Non-contrast CT images were reconstructed in 3-mm axial sections with a reconstruction kernel B35f. Contrast-enhanced images were reconstructed in 0.75-mm axial sections with a reconstruction kernel I30f by using a sinogram-affirmed iterative reconstruction algorithm. For all scans, the whole chest field-of-view was reconstructed in 1 mm axial sections within the acquired standard CCTA scan range, from the tip of the left apex to just above the coronary artery ostia, using a soft tissue kernel I30f and a lung kernel I70f.

### Evaluation and categorization of findings on CCTA

**Fig. 2:**
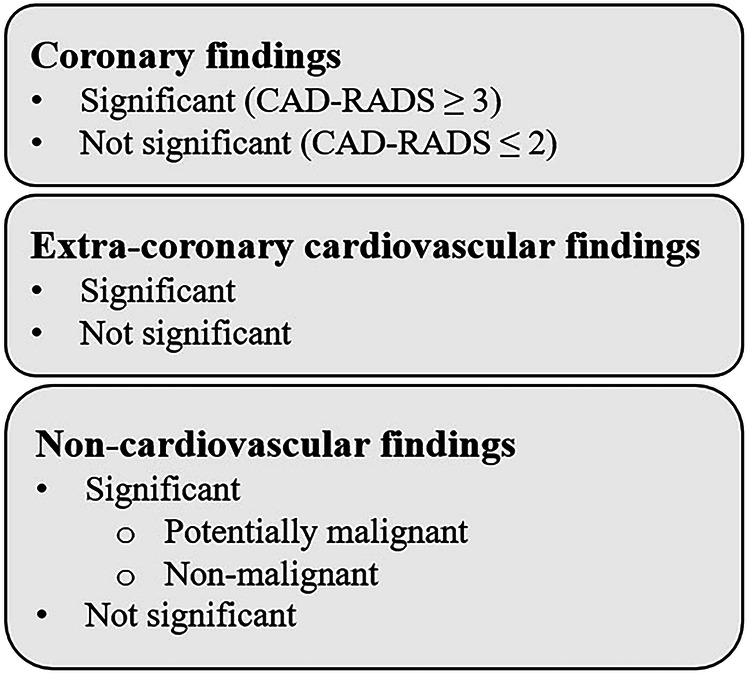
Classification scheme of findings on coronary computed tomography angiography (CCTA). Findings were categorized into coronary, extra-coronary cardiovascular, and non-cardiovascular findings, and stratified by clinical relevance. Non-cardiovascular findings were further subdivided into potentially malignant and non-malignant categories. Data were analyzed and reported at the patient level; patients could contribute to more than one subcategory

Radiology reports were systematically reviewed, and findings were grouped as coronary, extra-coronary cardiovascular, or non-cardiovascular. Coronary findings were assessed using coronary artery calcium score (CACS) [29, 30] and CAD-RADS classification [31, 32]; stenosis ≥ 50% (CAD-RADS ≥ 3) was considered significant (Fig. 2). Extra-coronary cardiovascular findings included valvular disease, vascular anomalies (aortic ectasia/aneurysm > 40/50 mm [33]), and pulmonary artery dilatation (> 30 mm [34]).

**Fig. 3:**
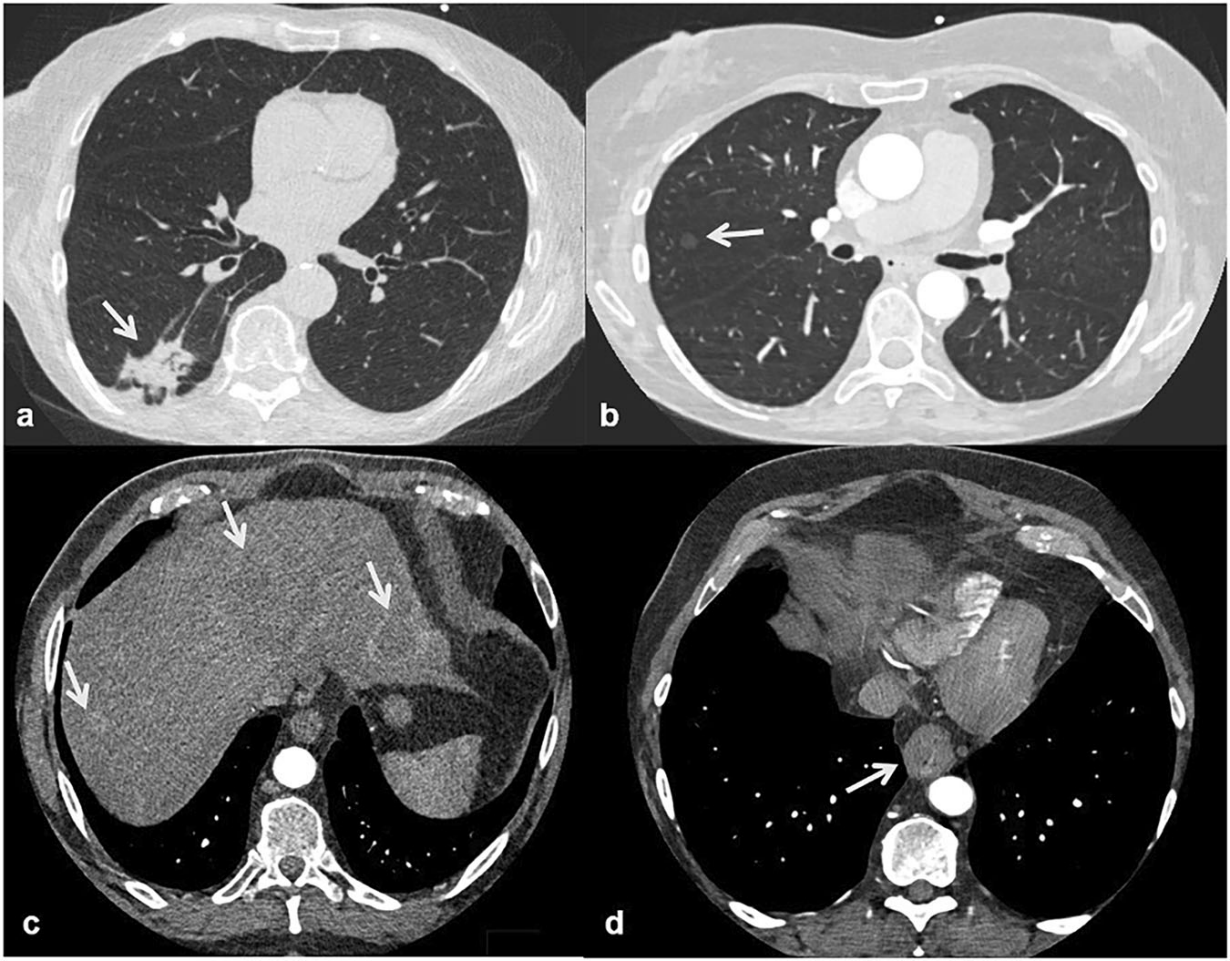
Examples of malignant extra-coronary findings detected on coronary computed tomography angiography (CCTA). **a** Pulmonary lesion confirmed as non-small cell lung cancer (NSCLC). **b** Pulmonary ground-glass nodule confirmed as NSCLC (adenocarcinoma subtype). **c** Liver lesions confirmed as metastases from pancreatic cancer. **d** Esophageal lesion was confirmed as esophageal adenocarcinoma

Non-cardiovascular findings encompassed all other incidental findings within the CCTA scan range not involving the heart or great vessels and were stratified as significant or not significant. Significant findings were further classified as potentially malignant or non-malignant. Potentially malignant findings were defined by radiologist recommendation for further work-up (e.g., biopsy or follow-up imaging, Fig. 3). Pulmonary nodules were followed up according to the Fleischner Society guidelines [35]; nodules with clearly benign characteristics, such as intrapulmonary lymph nodes, were not classified as potentially malignant. Mediastinal lymph nodes were classified by the radiologist, while lymphadenopathy was defined as a short-axis diameter of > 10 mm with round shape, heterogeneous density or irregular border.

Non-malignant significant findings included emphysema, liver steatosis or fibrosis, pulmonary embolism, bronchiectasis, interstitial lung disease (ILD), or pulmonary consolidations and ground-glass. Emphysema was visually assessed on non-contrast scans; hepatic steatosis was defined as liver attenuation < 40 Hounsfield Units [36, 37]. Findings considered to be of no clinical relevance and not requiring follow-up were categorized as not significant. All data were analyzed and reported at the patient level. In cases where individual patients presented with multiple findings across different subcategories (e.g., both pulmonary and mediastinal lesions), these were classified per patient, not per lesion. However, one patient could have findings in different categories. Subcategory counts are therefore not mutually exclusive.

### Follow-up and prevalence estimates

Patients with potentially malignant findings, pulmonary emphysema, or liver steatosis were followed up in the hospital's clinical record 3 years after CCTA. This included an assessment of follow-up imaging data in the local picture archiving and communication system (PACS, Sectra) and an evaluation of clinical history and follow-up consultations through the local hospital information system (Phoenix, CompuGroup Medical Schweiz AG). Prevalence estimates for significant findings were extrapolated to the full cohort by applying observed diagnostic rates in followed patients to those lost to follow-up, assuming equal probability of diagnosis. Exact 95% confidence intervals (CI) were calculated using the Clopper–Pearson method. A secondary sensitivity analysis stratified extrapolation by emphysema severity.

### Statistics

Categorical variables were reported as numbers and percentages, while continuous variables were reported as medians with interquartile ranges (IQRs). Comparisons between groups were performed using Fisher’s exact test for categorical variables and the Mann-Whitney U test for continuous variables. Binary logistic regression was used to evaluate the association between CACS groups (CACS = 0 vs. CACS > 0) and the presence of clinically significant findings. The dependent variable was the presence of the specific finding of interest, the independent variable was the CACS group, and adjustments were made for age and sex. Results are reported as odds ratios (OR) with 95% confidence intervals (CI). Statistical analysis was performed using statistical product and service solutions (SPSS) software (version 27; IBM).

## Results

### Baseline population

The study included 3295 patients, with baseline characteristics summarized in Table 1. The median age was 59 years; 1827 (55%) were male and 1468 (45%) female. A total of 1295 patients (39%) had no coronary artery calcification (CACS 0), while 2000 (61%) had CACS > 0. Patients with CACS > 0 were older and had higher rates of male sex, hypertension, diabetes, dyslipidemia, smoking history, and obesity (body mass index > 30 kg/m^2^).

**Table 1:** Baseline characteristics

	Total	CACS^a^ 0	CACS > 0	
	*n* (%)	*n* (%)	*n* (%)	*p*-value
Total	3295	1295 (39)	2000 (61)	
Female	1468 (45)	725 (56)	743 (37)	*p* < 0.01
Median age	59.4 (51.6–67.0^b^)	53.0 (44.8–61.0^b^)	62.9 (56.2–69.3^b^)	*p* < 0.01
Arterial hypertension	1325 (40)	385 (30)	940 (47)	*p* < 0.01
Diabetes mellitus	334 (10)	68 (5)	266 (13)	*p* < 0.01
Dyslipidaemia	1205 (37)	375 (29)	830 (42)	*p* < 0.01
History of smoking	907 (28)	287 (22)	620 (31)	*p* < 0.01
Family history of myocardial infarction or cardiovascular death	896 (27)	334 (26)	562 (28)	*p* = 0.15
Obesity (BMI^c^ > 30 kg/m^2^)	565 (17)	182 (14)	383 (19)	*p* < 0.01

Baseline characteristics of the study population, comparing patients without (CACS 0) and with (CACS > 0) coronary artery calcifications. Risk factors and clinical history data were extracted from the CT request form

^a^ Coronary artery calcification score (CACS)

^b^ Interquartile range (25th–75th percentile)

^c^ Body mass index

### Coronary findings and extra-coronary cardiovascular findings

Overall, 760 patients (23.1%) had CAD-RADS scores ≥ 3, indicating ≥ 50% stenosis. Among them, 16 patients (0.5%) had non-calcified plaques with CACS 0. High-grade stenosis (> 70%) was present in 323 patients (9.8%), with 285 (8.6%) CAD-RADS 4 and 38 (1.2%) CAD-RADS 5 (Table 2). Significant extra-coronary cardiovascular findings were observed in 931 patients (28.3%), mostly aortic ectasia (481, 14.6%) and pulmonary artery ectasia (181, 5.5%), both more frequent with CACS > 0 (*p* < .01). Other findings included atrial septal defects (3.3%), aortic valve degeneration (severe 0.3%; mild-moderate 13.5%), aortic atherosclerosis (severe 1.2%; mild-moderate 9.7%), and pericardial effusion > 10 mm (0.9%). Full details are in Table A1, Appendix A (electronic supplementary material).

**Table 2:** Primary results

	Total		CACS^a^ 0		CACS > 0			
	*n*	(%)	*n*	(%)	*n*	(%)	*p*-value	OR (95% CI)
Total	3295	(100)	1295	(100)	2000	(100)		
**Coronary findings**								
CACS 0	1295	(39.3)						
CACS > 0	2000	(60.7)						
CACS < 50th percentile	557	(16.9)			557	(27.9)		
CACS > 50th percentile	1376	(41.8)			1376	(68.8)		
Coronary stent	67	(2.0)			67	(3.4)		
CAD-RADS^b^ 0-2	2526	(76.7)	1279	(98.8)	1247	(62.4)		
CAD-RADS 3	437	(13.3)	10	(0.8)	427	(21.4)		
CAD-RADS 4	285	(8.6)	6	(0.5)	279	(14.0)		
CAD-RADS 5	38	(1.2)			38	(1.9)		
CAD-RADS N	9	(0.3)			9	(0.5)		
**Extra-coronary findings (total)**	3053	(92.7)	1150	(88.8)	1905	(95.3)	*p* = 0.09	1.3 (1.0–1.8)
Significant	1491	(45.3)	490	(37.8)	1001	(50.1)	*p* = 0.05	1.2 (1.0–1.4)
Not significant	2908	(88.3)	1081	(83.5)	1827	(91.4)	*p* = 0.35	1.1 (0.9–1.5)
**Extra-coronary cardiovascular findings**	1403	(42.6)	443	(13.4)	960	(29.1)	*p* = 0.04	1.2 (1.0–1.4)
Significant	931	(28.3)	291	(22.5)	640	(32.0)	*p* = 0.15	1.1 (1.0–1.4)
• Aortic ectasia^c^	481	(14.6)	129	(10.0)	352	(17.6)	*p* < 0.01	1.4 (1.1–1.9)
• Aortic aneurysm^d^	24	(0.7)	9	(0.7)	15	(0.8)	*p *= 0.03	1.2 (1.0–1.5)
• Pulmonary artery ectasia^e^	181	(5.5)	46	(3.6)	135	(6.8)	*p* < 0.01	1.6 (1.1–2.4)
• Atrial septal defect	109	(3.3)	62	(4.8)	47	(2.4)	*p* < 0.01	0.5 (0.3–0.7)
• Severe atherosclerosis	40	(1.2)	3	(0.2)	37	(1.9)	*p* = 0.02	4.2 (1.2–14.0)
Not significant	719	(21.8)	210	(16.2)	509	(25.5)	*p* = 0.02	1.3 (1.0–1.5)
**Non-cardiovascular findings**	2895	(87.9)	1081	(32.8)	1814	(55.1)	*p* = 0.49	1.1 (0.8–1.2)
Significant	833	(25.3)	266	(20.5)	567	(28.4)	*p* < 0.01	1.3 (1.1–1.5)
Potentially malignant	281	(8.5)	88	(6.8)	193	(9.7)	*p* = 0.17	1.2 (0.9–1.7)
• Pulmonary nodules/lesions	163	(4.9)	39	(3.0)	124	(6.2)	*p* < 0.01	1.7 (1.1–2.6)
• Mediastinal lymphadenopathy/lesions	26	(0.8)	11	(0.8)	15	(0.8)	*p* = 0.11	0.5 (0.2–1.2)
• Esophageal wall thickening/lesions	37	(1.1)	12	(0.9)	25	(1.3)	*p* = 0.27	1.6 (0.7–3.5)
• Liver lesions	50	(1.5)	22	(1.7)	28	(1.4)	*p* = 0.43	0.8 (0.4–1.5)
• Kidney lesions	1	(0.0)			1	(0.1)		
• Bowel wall thickening	3	(0.1)			3	(0.2)		
• Peritoneal lesions	1	(0.0)			1	(0.1)		
• Lytic bone lesions	7	(0.2)	2	(0.2)	5	(0.3)	*p* = 0.75	0.7 (0.1–4.4)
• Breast lesions	15	(0.5)	8	(0.6)	7	(0.4)	*p *= 0.82	0.9 (0.3–2.7)
Non-malignant	637	(19.3)	200	(15.4)	437	(21.9)	*p* = 0.06	1.2 (1.0–1.5)
• Pulmonary emphysema	352	(10.7)	85	(6.6)	267	(13.4)	*p* < 0.01	1.6 (1.2–2.1)
• Pulmonary infectious/inflammatory^f^	77	(2.3)	35	(2.7)	42	(2.1)	*p* = 0.76	1.1 (0.5–2.6)
• Bronchiectasis	21	(0.6)	6	(0.5)	15	(0.8)	*p* = 0.77	0.9 (0.3–2.4)
• Interstitial lung disease (ILD)	17	(0.5)	5	(0.4)	12	(0.6)	*p* = 0.71	0.8 (0.3–2.5)
• Pulmonary embolism	9	(0.3)	3	(0.2)	6	(0.3)	*p* = 0.82	0.8 (0.2–4.0)
• Liver steatosis^g^	147	(4.5)	65	(5.0)	82	(4.1)	*p* = 0.82	1.0 (0.7–1.4)
• Liver cirrhosis	9	(0.3)	2	(0.2)	7	(0.4)	*p* = 0.42	2.1 (0.4–12.1)
• Recent fractures	4	(0.1)	1	(0.1)	3	(0.2)		
Not significant	2816	(85.5)	1047	(80.8)	1769	(88.5)	*p* = 0.64	1.1 (0.8–1.3)

Primary imaging findings of coronary CT angiography (CCTA), stratified by presence of coronary artery calcification (CACS = 0 vs. CACS > 0). Coronary and extra-coronary findings are reported, along with associated odds ratios (OR) and 95% confidence intervals, adjusted for age and sex. All findings are reported at the patient level. Only the most frequent or statistically significant findings are shown. See Table A1 for detailed cardiovascular findings, Table A2 for non-significant non-cardiovascular findings, Table 3 for potentially malignant non-cardiovascular findings and follow-up, and Table 4 for non-malignant non-cardiovascular findings and follow-up

^a^ Coronary artery calcification score (CACS)

^b^ Coronary Artery Disease—Reporting and Data System

^c^ Aortic ectasia: root or ascending aorta > 40 mm

^d^ Aortic aneurysm: root or ascending aorta > 50 mm

^e^ Pulmonary artery ectasia: > 30 mm

^f^ Pulmonary consolidation, ground-glass opacity, tree-in-bud

^g^ Liver steatosis: liver < 40 Hounsfield Units in non-contrast scan

### Non-cardiovascular findings

Non-cardiovascular findings were reported in 2895 patients (87.9%), though most were not clinically significant, such as spinal degenerative changes (57.1%), low-suspicion pulmonary nodules (33.8%), bronchial wall thickening (14.2%), and hiatal hernia (11.8%; Table A2, Appendix A). Significant non-cardiovascular findings were present in 833 patients (25.3%), including 281 (8.5%) potentially malignant and 637 (19.3%) non-malignant findings. Among non-malignant findings, the most common were pulmonary emphysema (352, 10.7%), liver steatosis (147, 4.5%), and pulmonary consolidation/ground-glass (77, 2.3%; Table 2).

### Potentially malignant findings and follow-up

Potentially malignant findings were seen in 281 patients (8.5%), mainly pulmonary nodules (165, 5.0%), liver lesions (50, 1.5%), and esophageal wall thickening (37, 1.1%). Pulmonary nodules were associated with coronary calcification (*p* < 0.01); other findings were not. Three-year follow-up was available in 113 of 281 patients (40.2%) with potentially malignant findings, confirming malignancy in 32 patients (28.3%), of which 17/32 (53.1%) were previously unknown. Pulmonary malignancies were most frequent, followed by mediastinal, hepatic, and bone cancers (Table 3).

**Table 3:** Potentially malignant findings and follow-up (non-cardiovascular)

	Total		Follow up		Confirmed malignancy		New		Known	
	*n*	(%)	*n*	(%)	*n*	(%)^a^	*n*	(%)^b^	*n*	(%)^b^
Potentially malignant findings	281	(8.5)	113	(40.2)	32	(28.3)	17	(53.1)	15	(46.9)
Pulmonary lesions/nodules	165	(5.0)	71	(43.0)	15	(21.1)	13	(86.7)	2	(13.3)
• NSCLC^c^					11		9		2	
• Pulmonary carcinoid tumor					2		2			
• Metastases^d^					2		2			
Mediastinal lymphadenopathy/lesions	26	(0.8)	13	(50.0)	7	(26.9)	2	(28.6)	5	(71.4)
• Thymoma					1		1			
• Lymphoma					2				2	
• Metastases^e^					4		1		3	
Esophageal lesions/wall thickening	37	(1.1)	10	(27.0)	2	(20.0)	1	(50.0)	1	(50.0)
• Esophageal adenocarcinoma					2		1		1	
Liver lesions	50	(1.5)	19	(38.0)	6	(31.6)	2	(33.3)	4	(66.7)
• HCC^f^					1				1	
• Tubulopapillary neoplasia					1		1			
• Metastases^g^					4		1		3	
Kidney lesions	1	(0.0)	0	(0.0)						
Bowel wall thickening	3	(0.1)	0	(0.0)						
Peritoneal lesions	1	(0.0)	1	(100.0)	1	(100.0)			1	(100.0)
• Metastases^h^					1				1	
Lytic bone lesions	7	(0.2)	6	(85.7)	6	(100.0)			6	(100.0)
• Metastases^i^					6				6	
Breast lesions	15	(0.5)	7	(46.7)	1	(14.3)			1	(100.0)
• Breast cancer					1				1	

Follow-up outcomes in patients with potentially malignant findings on coronary CT angiography. Data include follow-up rates, proportion of confirmed malignancies, and proportion of newly diagnosed vs. previously known cancers. All data are reported at the patient level. Note that individual patients may have presented with multiple potentially malignant findings across different categories (e.g., both pulmonary and mediastinal lesions); therefore, numbers across subcategories are not mutually exclusive. The total number of confirmed malignancies refers to unique patients with at least one confirmed cancer diagnosis

^a^ Percentage among followed-up patients

^b^ Percentage among confirmed malignancies

^c^ Non-small cell lung cancer

^d^ Malignant melanoma, leiomyosarcoma of the uterus

^e^ NSCLC malignant melanoma, renal cell carcinoma (RCC), neuroendocrine tumor (NET) of the small bowel

^f^ hepatocellular carcinoma

^g^ RCC, pancreatic cancer, NET of the small bowel, acute myeloid leukemia

^h^ NET of the small bowel

^i^ Two prostate cancers, two multiple myelomas, RCC, NET of the small bowel

### Non-malignant findings and follow-up

Among 352 emphysema patients, 41 (11.6%) received pulmonology follow-up; COPD was diagnosed in 16/41 (39.0%), including 6/16 (37.5%) previously undiagnosed cases. Other pulmonary conditions included OSA (*n* = 5), asthma (*n* = 2), and CPFE (*n* = 1), with 3 previously unknown. In 17 of 41 patients (41.5%), no specific pulmonary diagnosis was obtained. Among 147 patients with significant liver steatosis, 10 (6.8%) had hepatology follow-up. Steatosis was confirmed in all cases, with 7 diagnosed as MASLD and 3 alcohol-related liver disease (ARLD). Liver fibrosis was present in three of these patients (Table 4).

**Table 4:** Significant non-malignant findings and follow-up (non-cardiovascular)

	*n*	(%)		*n*	(%)		*n*	(%)
**Total**	637	(19.3)^a^						
**Pulmonary**	487	(14.8)	**Liver**	158	(4.8)	**Other abdominal**	14	(0.4)
Emphysema	352	(10.7)	Liver steatosis	147	(4.5)	Splenomegaly	7	(0.2)
Follow up	41	(11.6)^b^	Follow up	10	(6.8)^b^	Splenic infarctions	4	(0.1)
• COPD^d^	16	(39.0)^c^	• MASLD^k^	*7*	(70.0)^c^	Upside down stomach	3	(0.1)
*•* OSA^e^	5	(12.2)^c^	* •* With fibrosis	2				
*•* Asthma	2	(4.9)^c^	• ARLD^l^	3	(30.0)^c^	**Skeletal/soft tissues**	17	(0.5)
*•* CPFE^f^	1	(2.4)^c^	• With fibrosis	1		Recent fractures	4	(0.1)
• No conclusive diagnosis	17	(41.5)^c^	Liver cirrhosis	9	(0.3)	Inflammatory skeletal changes	7	(0.2)
Tree-in-bud pattern	45	(1.4)	• Autoimmune	1		Asymmetric gynecomastia	5	(0.2)
Consolidation/ground-glass	32	(1.0)	*•* Hepatitis C virus	1				
Interstitial lung disease^g^	17	(0.5)	*•* Metabolic associated	2				
• IPF^h^	2		• Alcoholic	5		**Miscellaneous**	1	(0.0)
*•* Systemic sclerosis	3		Ascites	5	(0.2)	Dislocated catheter in left pulmonary artery	1	(0.0)
• Anti-synthetase syndrome	2		• In liver cirrhosis	4				
• GPA^i^	1		• In AML^m^ with liver infiltration	1				
• Sarcoidosis	2		Portosystemic shunts	5	(0.2)			
• PPFE^j^	1		• In liver cirrhosis	3				
• Not classified	6							
Bronchiectasis	21	(0.6)						
• Basal predominance	13							
• Multifocal	8							
Pneumothorax	1	(0.0)						
Pulmonary embolism	9	(0.3)						
Pleural effusion	46	(1.4)						

Patients with significant non-malignant findings. Results are stratified by organ system and final diagnoses, where available. All findings are reported at the patient level. For pulmonary emphysema and liver steatosis, follow-up rates and diagnostic outcomes among patients with follow-up are additionally reported

^a^ Percentage among all patients (3295) if not mentioned otherwise

^b^ Percentage of follow-up

^c^ Percentage among followed-up patients

^d^ Chronic obstructive pulmonary disease

^e^ Obstructive sleep apnea

^f^ Combined pulmonary fibrosis and emphysema

^g^ Combinations of reticulation, ground-glass, traction bronchiectasis and honey-combing

^h^ Idiopathic pulmonary fibrosis

^i^ Granulomatosis with polyangiitis

^j^ Pleuroparenchymal fibroelastosis

^k^ Metabolic dysfunction-associated steatotic liver disease

^l^ Alcohol-related liver disease

^m^ Acute myeloid leukemia

### Prevalence estimates of significant non-cardiovascular findings

Extrapolating follow-up results to the entire cohort suggests that up to 80 patients (2.4%; 95% CI: 1.8–3.2%) may have harbored malignancies, including 42 with previously unknown cases, mostly lung cancer (*n* = 35; Table A3, Appendix A). Assuming no malignancies among patients lost to follow-up, the minimum prevalence would be 1.0%.

For non-malignant conditions, extrapolated estimates suggest 137 patients with COPD (4.2%; 95% CI: 2.6–5.9%), including 52 newly diagnosed (1.6%; 95% CI: 0.6–3.1%). Asthma or OSA could account for up to 60 additional cases (1.8%; 95% CI: 0.8–3.4%), with 26 previously undiagnosed (0.8%; 95% CI: 0.2–2.1%). The estimated prevalence of MASLD- and ARLD-related steatosis was 4.5% (95% CI: 3.1–4.5%), with 1.3% showing liver fibrosis in follow-up (95% CI: 0.5–1.2%). Among patients without steatosis, 0.4% showed imaging signs of liver cirrhosis (e.g., fibrotic liver remodeling, ascites, or portosystemic collaterals). Among the 37 patients with esophageal wall thickening, 10 (27.0%) underwent follow-up, identifying 2 cases of esophageal adenocarcinoma and 8 non-malignant diagnoses. Extrapolated to the full cohort, up to 1.1% (95% CI: 0.3–2.8%) may have clinically significant esophageal conditions.

## Discussion

This study demonstrates that clinically significant extra-coronary findings are common in CCTA. Among 3295 patients, 23.1% had a coronary stenosis ≥ 50% (CAD-RADS score ≥ 3), 28.3% had significant extra-coronary cardiovascular findings, and 25.3% had significant non-cardiovascular findings. Of 281 patients (8.5%) with potentially malignant findings, 113 (40.2%) underwent follow-up, confirming malignancy in 32 (28.3% of those with follow-up). Extrapolated malignancy prevalence was 2.4% (95% CI: 1.8–3.2%), with a conservative minimum estimate of 1.0%.

Significant non-malignant non-cardiovascular findings were identified in 637 patients (19.3%), with pulmonary emphysema in 352 patients (10.7%) and significant liver steatosis in 147 patients (4.5%) being the most common ones. Based on extrapolated prevalence rates, 4.2% of CCTA patients may have COPD, which is previously unrecognized in 1.6% of all CCTA patients, while another 1.8% CCTA patients may have asthma and OSA, potentially previously unrecognized in 0.8% of all CCTA patients. Knowing that dyspnea and chest tightness are symptoms that may not be related to CAD, but also to COPD [38], asthma, or OSA [39], a diagnosis of pulmonary emphysema with follow-up by a pulmonologist is important. Similarly, symptoms such as fatigue, weakness, and upper abdominal discomfort could indicate MASLD or ARLD [40], affecting approximately 4.5% CCTA patients, including 1.3% with liver fibrosis. These findings highlight the need for increased awareness among referring cardiologists and general practitioners regarding the prevalence of potentially significant malignant and non-malignant non-cardiovascular findings in CCTA. The prevalence of those findings is comparable to that of high-grade coronary artery stenoses (CAD-RADS score 4: 285, 8.6%; CAD-RADS score 5: 38, 1.2%).

This underscores the critical role of radiologists in the comprehensive evaluation of both coronary and extra-coronary findings. Given the high frequency of extra-coronary findings (92.7%) in CCTA patients, a careful assessment is essential to avoid unnecessary follow-up while ensuring clinically relevant findings are addressed. The American College of Radiology (ACR) Incidental Findings Committee has published recommendations on managing mediastinal and cardiovascular findings on thoracic CT [41], and incorporating such structured approaches into CCTA workflows may improve decision-making and outcomes. Radiologists’ expertise is vital for distinguishing significant incidental findings from benign ones, requiring not only strong cardiac imaging skills but also familiarity with extra-cardiac pathology. Previous research shows that additional training can significantly enhance the detection of incidental findings. For example, Verdini et al [42] demonstrated that structured training improved radiologists’ ability to identify cardiac abnormalities on routine chest CT scans. These findings highlight the importance of ongoing education and training to ensure diagnostic accuracy and appropriate follow-up in CCTA.

The results align with previous studies on the prevalence of extra-cardiovascular findings. A systematic review by Kay et al reported a median prevalence of extracardiac findings of 45% (range 7–100%) and clinically significant findings of 17% (range 1–67%) [21]. A recent analysis from the European Society of Cardiovascular Radiology MR/CT Registry (over 200,000 cardiac CT exams) found a prevalence of relevant extracardiac findings of 3.3%, with pulmonary lesions and nodules comprising the largest share (28.7%) [25]. A systematic review and meta-analysis by Flor et al reported a 0.7% prevalence of previously undiagnosed malignancies on cardiac CT, over 70% of which were lung cancers [43]. Our findings fall within these ranges and add new insights into their clinical relevance. Notably, pulmonary emphysema and potentially malignant pulmonary nodules were more common in patients with coronary calcification. A possible association between lung cancer and coronary artery calcification was observed: 12 of 13 confirmed lung cancer cases had CACS > 0 (*p* = 0.09, OR 0.7–47.1), indicating an overlap of risk factors for coronary artery disease and lung cancer. These findings support the potential value of including full chest CT in high-risk CCTA patients, particularly smokers, to improve early lung cancer detection. In the NELSON trial—one of the largest lung cancer screening studies—the detection rate was 0.9% (203 cancers among 22,600 patients) [28], comparable to the extrapolated 1.0% rate in our CCTA cohort. Further research should explore the benefit of combined CCTA and chest screening in high-risk patients and evaluate the role of CCTA in future lung cancer screening strategies.

This retrospective, single-centre study has limitations regarding generalizability and potential variability in radiological interpretation. Extrapolation of confirmed diagnoses to the full cohort, assuming missing-at-random follow-up, introduces potential selection bias and may overestimate true prevalence—especially for COPD extrapolated from CT-detected emphysema. To address this, we explored a severity-stratified extrapolation in a secondary analysis (Table A3, Appendix), which yielded slightly different prevalence estimates. However, we opted not to apply this approach in the primary analysis in order to preserve methodological consistency and comparability across findings. At the same time, airway-predominant COPD [44, 45] may be underestimated due to qualitative reporting of bronchial wall thickening. The low prevalence of potentially malignant mediastinal lymphadenopathy (0.8%) may be underestimated due to radiologist judgment and limited scan range. Finally, the study did not assess epicardial adipose tissue (EAT), an emerging cardiovascular risk biomarker [46]. Prospective studies with standardized follow-up, quantitative CT metrics, dedicated mediastinal protocols, and EAT assessment are needed.

In conclusion, both coronary and extra-coronary findings are common within the scan range of CCTA and carry important clinical implications. Careful management of these findings is necessary, and additional screening protocols may be beneficial for high-risk patients.

## Supplementary information

Supplementary information

## Abbreviations

ARLD: Alcohol-related liver disease
CACS: Coronary artery calcium score
CAD: Coronary artery disease
CAD-RADS: Coronary Artery Disease—Reporting and Data System
CCTA: Coronary computed tomography angiography
COPD: Chronic obstructive pulmonary disease
MASLD: Metabolic dysfunction-associated steatotic liver disease
NSCLC: Non-small cell lung cancer
OSA: Obstructive sleep apnea
