# Supplementary material for: Beyond the heart: prevalence and implications of extra-coronary findings in coronary CT angiography: a retrospective study
Source: Eur Radiol. 2025 Aug 5;36(2):1120–30. doi: 10.1007/s00330-025-11886-6 (PMC12953475; doi:10.1007/s00330-025-11886-6)
Supplement: Supplementary file 1 — Supplementary information [file 330_2025_11886_MOESM1_ESM.pdf]

Beyond the Heart: Prevalence and Implications of Extra-Coronary Findings in Coronary CT Angiography – A Retrospective Study

ELECTRONIC SUPPLEMENTARY MATERIAL

Appendix Tables A1-3

Table A1. Extra-coronary cardiovascular findings

	n	%
**Total**	1403	42.6
**Significant**	931	28.3
Pericardium		
Pericardial effusion >10mm	31	0.9
Pericardial thickening	6	0.2
Heart valves		
Severe AV[a] degeneration	9	0.3
Bicuspid AV	22	0.7
AV vegetations	20	0.6
Severe MA[b]calcification	19	0.6
MV[c] leaflet thickening/calcification	43	1.3
MV vegetations	7	0.2
Ventricles		
Dilated left ventricle	10	0.3
Left ventricular hypertrophy	29	0.9
Pulmonary congestion	24	0.7
Ischemic myocardial lesion	17	0.5
Left ventricular aneurysm	4	0.1
Left ventricular thrombus	5	0.2
Dilated right ventricle	18	0.5
Ventricular septum defect	3	0.1
Atria		
Dilated left atrium	37	1.1
Dilated right atrium	30	0.9
Left atrial appendage flow artefact	15	0.5
Left atrial tumor	3	0.1
Atrial septum defect	109	3.3
Vessels		
Severe atherosclerosis	40	1.2
Aortic ectasia[d]	481	14.6
40-44mm	384	79.8
45-49mm	97	20.2
Aortic aneurysm[e]	24	0.7
Aortic dissection	23	0.7
Stanford Type A	14	60.9
Stanford Type B	9	39.1
Pulmonary artery ectasia (>30mm)	181	5.5
30-34mm	149	82.3
35-39mm	31	17.1

40-44mm	1	0.6
**Not significant**	719	21.8
Pericardial calcification	9	0.3
Pericardial cyst	6	0.2
Mild to moderate AV degeneration	445	13.5
Mild to moderate MA calcification	111	3.4
Left atrial diverticulum	202	6.1
Atrial septum pouch	128	3.9
Atrial septum aneurysm	31	0.9
Mild to moderate Atherosclerosis	319	9.7
Common ostium of pulmonary veins	28	0.8
Left superior vena cava	3	0.1
Arteria lusoria	1	0.0

Table A1. Comprehensive list of cardiovascular extra-coronary findings identified on coronary computed tomography angiography (CCTA), stratified by clinical significance

[a] aortic valve; [b] mitral annulus; [c] mitral valve; [d] sinus of Valsalva or ascending aorta >40 mm; [e] sinus of Valsalva or ascending aorta >50 mm

Table A2. Not significant non-cardiovascular findings

	(n)	(%)		(n)	(%)
**Total**	2816	86.0	**Abdomen**	332	10.1
**Pulmonary**	2136	64.8	Benign hepatic lesion[b]	326	9.9
Low suspicion pulmonary nodules[a]	1114	33.8	Benign splenic lesion[c]	12	0.4
Focal scarring/bronchiectasis	130	3.9	Benign kidney lesion[d]	15	0.5
Dystelectasis/focal atelectasis	1171	35.5	Adrenal incidentaloma[e]	5	0.2
Pleural plaques/calcifications	51	1.5	Cholecystolithiasis	6	0.2
Bronchial wall thickening	469	14.2	Hiatal hernia	389	11.8
Tracheal/bronchial sclerosis	70	2.1	Diaphragmatic hernia	44	1.3
Bullae/ cysts	100	3.0	Colon diverticulosis	7	0.2
**Mediastinal**	100	3.0	**Skeletal**	1958	59.4
Reactive lymph nodes	35	1.1	Benign bone lesion[f]	65	2.0
Lymph node calcifications	36	1.1	DISH[g]	48	1.5
Persisting thymus	29	0.9	Schmorl's nodes	32	1.0
**Soft tissue**	8	0.2	Mild to moderate degenerative spine changes	1823	55.3
Probably benign nodule	4	0.1			
Lipoma	5	0.2	Severe degenerative spine changes	59	1.8
**Gynecological**	162	4.9			
Breast macrocalcifications	52	1.6	Osteopenia	226	6.9
Gynecomastia	57	1.7	Scoliosis	20	0.6

Table A2. Summary of non-cardiovascular extra-coronary findings considered clinically non-significant. Findings are grouped by anatomical region

[a] <6 mm, granuloma, lymph nodes, previously known, no recommendation for follow-up by radiologist; [b] cyst, hemangioma, FNH, AVM, shunt, calcifications; [c] cyst, hemangioma, calcifications; [d] cyst, focal scarring; [e] adenoma, myelolipoma; [f] hemangioma, enostosis; [g] diffuse idiopathic skeletal hyperostosis

Table A3. Extrapolation of significant non-cardiovascular findings

	Total	FU	Dx	Extrapolation to all patients	New	Extrapolation to all patients
	n (%)[a]	n (%)[b]	n (%)[c]	%(CI)[a]	n (%)[d]	%(CI)[a]
**Emphysema, total**	352 (10.7)	41 (11.6)				
COPD[e]			16 (39.0)	4.2 (2.6-5.9)	6 (37.5)	1.6 (0.6-3.1)
Other diagnoses			8 (19.5)	2.1 (0.9-3.6)	4 (50.0)	1.0 (0.3-2.7)
OSA[f]			5 (12.2)	1.3 (0.4-2.8)	2 (40.0)	0.5 (0.1-1.5)
Asthma			2 (4.9)	0.5 (0.1-1.8)	1 (50.0)	0.3 (0.0-1.4)
CPFE[g]			1 (2.4)	0.3 (0.0-1.4)	1 (100.0)	0.3 (0.0-1.4)
No conclusive diagnosis			17 (41.5)			
**Emphysema, mild/ moderate**	318 (9.7)	33 (10.4)				
COPD			9 (27.3)	2.6 (1.3-4.4)	3 (33.3)	0.9 (0.3-2.4)
Other diagnoses			8 (24.2)	2.3 (1.0-4.0)	4 (50.0)	1.2 (0.4-2.9)
OSA			5 (15.2)	1.5 (0.5-3.1)	2 (40.0)	0.6 (0.2-1.7)
Asthma			2 (6.1)	0.9 (0.1-1.9)	1 (50.0)	0.3 (0.0-1.4)
CPFE			1 (3.0)	0.3 (0.0-1.5)	1 (100.0)	0.3 (0.0-1.4)
No conclusive diagnosis			16 (48.5)			
**Emphysema, severe**	34 (1.0)	8 (23.5)				
COPD			7 (87.5)	0.9 (0.5-1.0)	3 (42.9)	0.4 (0.1-1.3)
No conclusive diagnosis			1 (12.5)			
**Potentially malignant findings**	281 (8.5)	113 (40.2)				
Confirmed			32 (28.3)	2.4 (1.8-3.2)	17 (53.1)	1.3 (0.9-1.7)
Pulmonary lesions/ nodules	165 (5.0)	71 (43.0)				
Confirmed			15 (21.1)	1.1 (0.7-1.6)	13 (86.7)	0.9 (0.6-1.3)
Mediastinal lymphadenopathy/ lesions	26 (0.8)	13 (50.0)				
Confirmed			7 (53.8)	0.4 (0.2-0.6)	2 (28.6)	0.1 (0.0-0.3)
Liver lesions	50 (1.5)	19 (38.0)				
Confirmed			6 (31.6)	0.5 (0.2-0.8)	2 (33.3)	0.2 (0.0-0.4)
Lytic bone lesions	7 (0.2)	6 (85.7)				
Confirmed			6 (100.0)	0.2 (0.1-0.2)		
Breast lesions	15 (0.5)	7 (46.7)				
Confirmed			1 (14.3)	0.1 (0.0-0.2)		
**Liver steatosis**	147 (4.5)	10 (6.8)				

MASLD[h]			7 (70.0)	3.1 (1.6-4.2)	6 (85.7)	2.7 (1.6-3.6)
With fibrosis			2 (20.0)	0.9 (0.3-1.5)	1 (50.0)	0.5 (0.1-1.2)
ARLD[i]			3 (30.0)	1.3 (0.3-2.9)	1 (33.3)	0.4 (0.1-1.1)
With fibrosis			1 (10.0)	0.4 (0.0-0.5)		0.2 (0.0-0.5)
**Esophageal wall thickening**	37 (1.1)	10 (27.0)				
Adenocarcinoma			2 (20.0)			
Reflux esophagitis			3 (30.0)	0.3 (0.1-0.8)	2 (66.7)	0.2 (0.0-0.6)
Chronic gastritis			2 (20.0)	0.2 (0.0-0.6)	2 (100.0)	0.2 (0.0-0.6)
H. pylori gastritis			1 (10.0)	0.1 (0.0-0.5)	1 (100.0)	0.1 (0.0-0.5)
Duplication cyst			1 (10.0)	0.1 (0.0-0.5)	1 (100.0)	0.1 (0.0-0.5)
Esophageal varices			1 (10.0)	0.1 (0.0-0.5)	1 (100.0)	0.1 (0.0-0.5)

Table A3. This table summarizes follow-up (FU) rates, diagnostic outcomes (Dx), and extrapolated prevalence estimates for significant non-cardiovascular findings in coronary CT angiography (CCTA) patients. All data are reported at the patient level. “Dx” refers to the final diagnosis determined through follow-up. “New” indicates whether the diagnosis was newly established or already known at the time of imaging. Prevalence estimates were extrapolated by applying the proportion of confirmed diagnoses among patients with follow-up to the entire cohort of patients with the respective finding, assuming equal probability of diagnosis in patients lost to follow-up. Exact 95% confidence intervals for the extrapolated prevalence were calculated using the Clopper–Pearson method for binomial proportions

[a] percentage among all patients (3295); [b] percentage of follow up; [c] percentage among followed up patients; [d] percentage among respective diagnosis group; [e] chronic obstructive pulmonary disease; [f] obstructive sleep apnea syndrome; [g] combined pulmonary fibrosis and emphysema; [h] metabolic-dysfunction associated steatotic liver disease; [i] alcohol-related liver disease
